# Re-sedation using remimazolam anesthesia in patients with multiple injuries during recovery: a case report and literature review

**DOI:** 10.3389/fmed.2025.1702891

**Published:** 2026-01-07

**Authors:** Guozun He, Chuangguo Shi, Wei Gao

**Affiliations:** 1 Department of Anesthesiology, Xi' an Aerospace Hospital Affiliated to Northwest University, Xi’an, Shaanxi, China; 2 Department of Anesthesiology and Center for Brain Science and Center for Translational Medicine, The First Affiliated Hospital of Xi’an Jiaotong University, Xi’an, Shaanxi, China

**Keywords:** remimazolam besylate, re-sedation, flumazenil, case report, general anesthesia

## Abstract

Remimazolam combined with flumazenil can shorten recovery time, but the occurrence of re-sedation may put the patient at risk. Herein, we report a case of a 50-year-old woman who underwent general anesthesia using remimazolam. During emergence from general anesthesia, she briefly regained consciousness after receiving 0.4 mg of flumazenil and then fell unconscious again. The disturbance of consciousness lasted for 75 min. We diagnosed re-sedation after ruling out other possible causes. We simulated anesthetic concentration changes using TIVA trainer software, further confirming re-sedation through mechanistic analysis. Additionally, we reviewed the literature and analyzed the potential reasons for the occurrence of re-sedation under various conditions. The occurrence of re-sedation is not only related to the administration of flumazenil but also to individual differences in the effect-site concentration of remimazolam and the phenomenon of rapid tolerance. In clinical practice, flumazenil should be used cautiously, avoiding single high-dose administration, and considering delayed administration when appropriate. For critically ill patients, clinicians should closely monitor and guard against the occurrence of re-sedation. Further research is needed to determine the optimal time to administer flumazenil and to identify the demographic and clinical characteristics of patients who experience re-sedation, thereby guiding patient safety.

## 1 Introduction

Behavioral disturbances in the perioperative period exhibit heterogeneous clinical presentations and can greatly impact the patient’s overall medical condition. Hypoactive emergence, which is characterized by lethargy and hypovigilance, is associated with a longer stay in the post-anesthesia care unit and an increased length of hospital stay (1). The application of drugs with a rapid onset and efficient metabolism may reduce the occurrence of incomplete recovery. Remimazolam is an ultra-short-acting intravenous benzodiazepine characterized by rapid metabolism, minimal accumulation, and stable hemodynamics, making it suitable for anesthesia induction and the maintenance of elderly or critically ill patients (2–4). The specific antagonist of remimazolam, flumazenil, can accelerate the recovery process after general anesthesia. However, their combined use carries a high risk of re-sedation (5). Re-sedation, a type of hypoactive emergence, is defined as a decrease in the Richmond Agitation-Sedation Scale of at least 1 (6). Re-sedation is likely to put the patient in danger, as the anesthesiologist may relax his vigilance after the patient wakes up.

The prevailing mechanism of re-sedation primarily suggests that as flumazenil is metabolized, the remaining remimazolam rebinds with the receptors, thereby causing re-sedation (7). However, the occurrence of re-sedation as reported seems to be more complex. In current research, re-sedation is typically reported in the form of case reports or adverse reactions, and systematic research is lacking. Understanding the mechanism of re-sedation is of great significance to the perioperative safety management of patients.

This report aims to present a clinically significant case of re-sedation following the reversal of remimazolam using flumazenil, analyze its potential mechanisms, and discuss implications for safe anesthetic practice. We conducted this case report in line with the CAse REport (CARE) guidelines. Written informed consent was obtained from the patient for the publication of this case report and any accompanying images.

## 2 Case description

**Figure 1:**
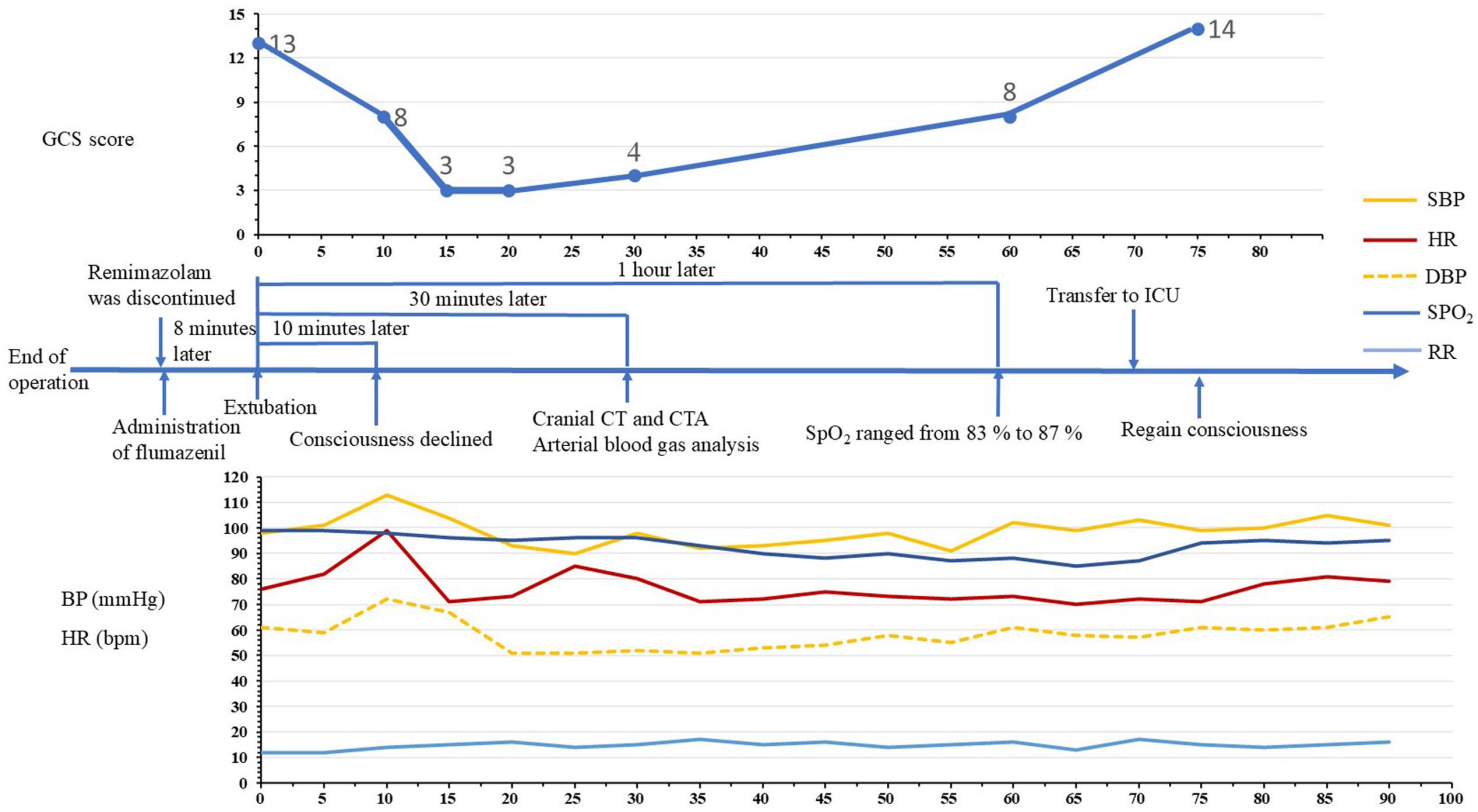
Time course of re-sedation. BP, blood pressure; SBP, systolic blood pressure; DBP, diastolic blood pressure; HR, heart rate; GCS, Glasgow Coma Scale; ICU, intensive care unit; SpO_2_, pulse oxygen saturation.

A 50-year-old woman (160 cm height; 60 kg body weight) with no significant medical history was admitted to our hospital due to a closed chest injury and humeral fracture. Emergency surgery was performed to stabilize multiple rib fractures and repair a ruptured lung. The anesthesia method was a paravertebral nerve block (0.3% ropivacaine hydrochloride, 15 mL) combined with general anesthesia (maintenance included remifentanil, remimazolam, and cisatracurium besylate). Due to the administration of mechanical ventilation to assist breathing after the surgery, and the removal of the tracheal tube on the third day after the operation, it was difficult to determine whether re-sedation had occurred. One week later, the patient underwent a second operation under general anesthesia to treat the right humeral fracture. The patient had mild anemia and hypoproteinemia, and due to atelectasis, a fiberoptic bronchoscopy was also performed (Supplementary Table 1; Figure 1). During emergence from general anesthesia, the patient briefly regained consciousness before experiencing further consciousness disturbance.

Standard monitoring was initiated upon the patient’s entry into the operating room. The patient’s vital signs were as follows: blood pressure (BP) 113/75 mmHg, heart rate (HR) 85 bpm, and pulse oxygen saturation (SpO_2_) 90% (room air). After ultrasound-guided right brachial plexus nerve block (interosseous groove, 0.3% ropivacaine 20 mL), anesthesia was induced with sufentanil 30 μg, etomidate 18 mg, and cisatracurium besylate 9 mg; an endotracheal tube was placed 2 min later. Anesthesia maintenance included remifentanil (0.05–0.1 μg/kg/min), remimazolam besylate (1 mg/kg/h, continuously infused until the end of surgery), and dexmedetomidine (DEX; 0.4 μg/kg/h, discontinued 1 h before the end of surgery). No other opioid drugs were administrated during the surgery. The duration of anesthesia was 3 h and 10 min (Supplementary Figure 2). During the emergence phase, remimazolam infusion was discontinued with simultaneous administration of 0.4 mg flumazenil. Approximately 8 min later, the patient resumed spontaneous breathing with a tidal volume of 300 mL. Neostigmine 1 mg, atropine 0.5 mg was administrated to antagonize residual neuromuscular blockade. She opened her eyes in response to tactile stimulation, and the tracheal tube was removed following adequate sputum suction. The patient’s STEWARD score was 5, and her Glasgow Coma Scale (GCS) score was 13 (Eye-opening 3, Verbal response 4, Motor response 6). The patient was transferred to the post-anesthesia care unit for continuous oxygen inhalation (oxygen flow rate, 2 L/min) and standard monitoring.

However, the patient gradually developed impaired consciousness (Figure 1). Ten minutes after extubation, the patient’s level of consciousness declined, and her GCS score dropped to 8 (Eye-opening 2, Verbal response 2, Motor response 4). Fifteen minutes after extubation, her level of consciousness further deteriorated, with a GCS score of 3 (Eye-opening 1, Verbal response 1, Motor response 1). Vital signs were as follows: SpO_2_ 96%, HR 85 bpm, and BP 90/51 mmHg. Twenty minutes after extubation, there was no significant improvement in the patient’s consciousness; her SpO_2_ was 96%, HR was 80 bpm, BP was 98/52 mmHg, and her GCS score remained at 3. Physical examination revealed pinpoint pupils with a mildly delayed light reflex. Thirty minutes after extubation, her SpO_2_ gradually decreased. Despite intermittent back percussion, the patient’s SpO_2_ level remained between 90 and 93%; her GCS score improved to 4 (Eye-opening 1, Verbal response 1, Motor response 2). Arterial blood gas analysis showed the following: pH 7.39, PaO_2_: 74.00 mmHg, PaCO_2_: 42.3 mmHg, Na^+^: 147 mmol/L, K^+^: 4.27 mmol/L, AG: 20.6 mmol/L, BE: 0.3 mmol/L, and Lac: 0.80 mmol/L. The neurological examination revealed a positive bilateral Babinski sign. Brain computed tomography and computed tomography angiography revealed no abnormalities (Supplementary Figure 3). One hour after extubation, the patient’s consciousness had improved slightly, with a GCS score of 8 (Eye-opening 2, Verbal response 2, Motor response 4). However, her SpO_2_ ranged from 83 to 87%, and sputum aspiration and dorsal percussion did not result in a significant improvement. After being transferred to the intensive care unit (ICU) and receiving high-flow nasal oxygen for 5 min, her awareness had improved considerably by 75 min after extubation. Her GCS score was 14 (Eye-opening 3, Verbal response 5, Motor response 6), and she reported no adverse memories of the entire process. Three days after surgery, the patient was transferred to the general ward and was discharged 2 weeks later.

## 3 Literature review

A systematic literature search was conducted in Medline/PubMed and Embase databases up to July 30, 2024, using the keywords “remimazolam” and “re-sedation.” The search was restricted to English-language articles and human studies. Among the retrieved records, re-sedation events were reported in three case reports and four clinical studies. The incidence of re-sedation in clinical studies ranges from 2 to 22%, and it occurs 30 to 45 min after flumazenil antagonism. Neither a single high dose nor a divided low dose of flumazenil prevented re-sedation. In case reports, the earliest re-sedation occurred 13 min after flumazenil antagonism, the latest occurred 6 h and 35 min after antagonism, and the most extended duration of re-sedation was 12 h. All these patients eventually awoke safely, but this highlights the complexity of the mechanisms involved in re-sedation.

## 4 Discussion

When combined with flumazenil, remimazolam can facilitate rapid recovery of consciousness, but it does not improve patient safety (8). The occurrence of re-sedation may be life-threatening and increase medical expenses. Patients should be monitored for a sufficient period after antagonism to prevent the occurrence of re-sedation (9).

**Figure 2:**
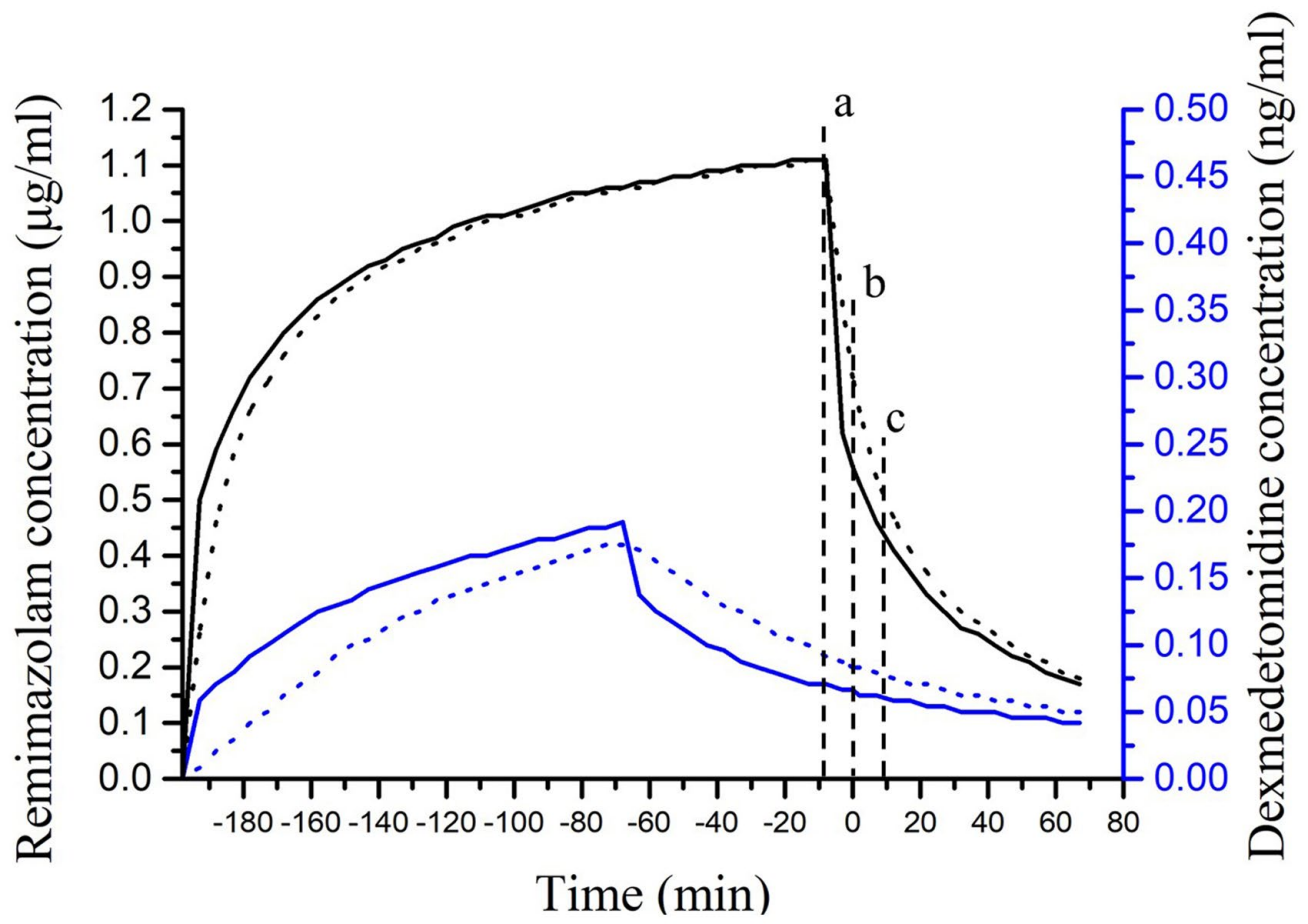
Trends in drug concentrations of remimazolam and DEX. The solid curve represents plasma concentrations, and the dashed curve represents effect-site concentrations. Remimazolam (black curve) is referenced from the left *Y*-axis; DEX (blue curve) is referenced from the right *Y*-axis. Vertical dashed lines correspond to case events as follows: (a) Remimazolam infusion stopped. (b) The time of extubation. (c) Decline in consciousness noted.

**Figure 3:**
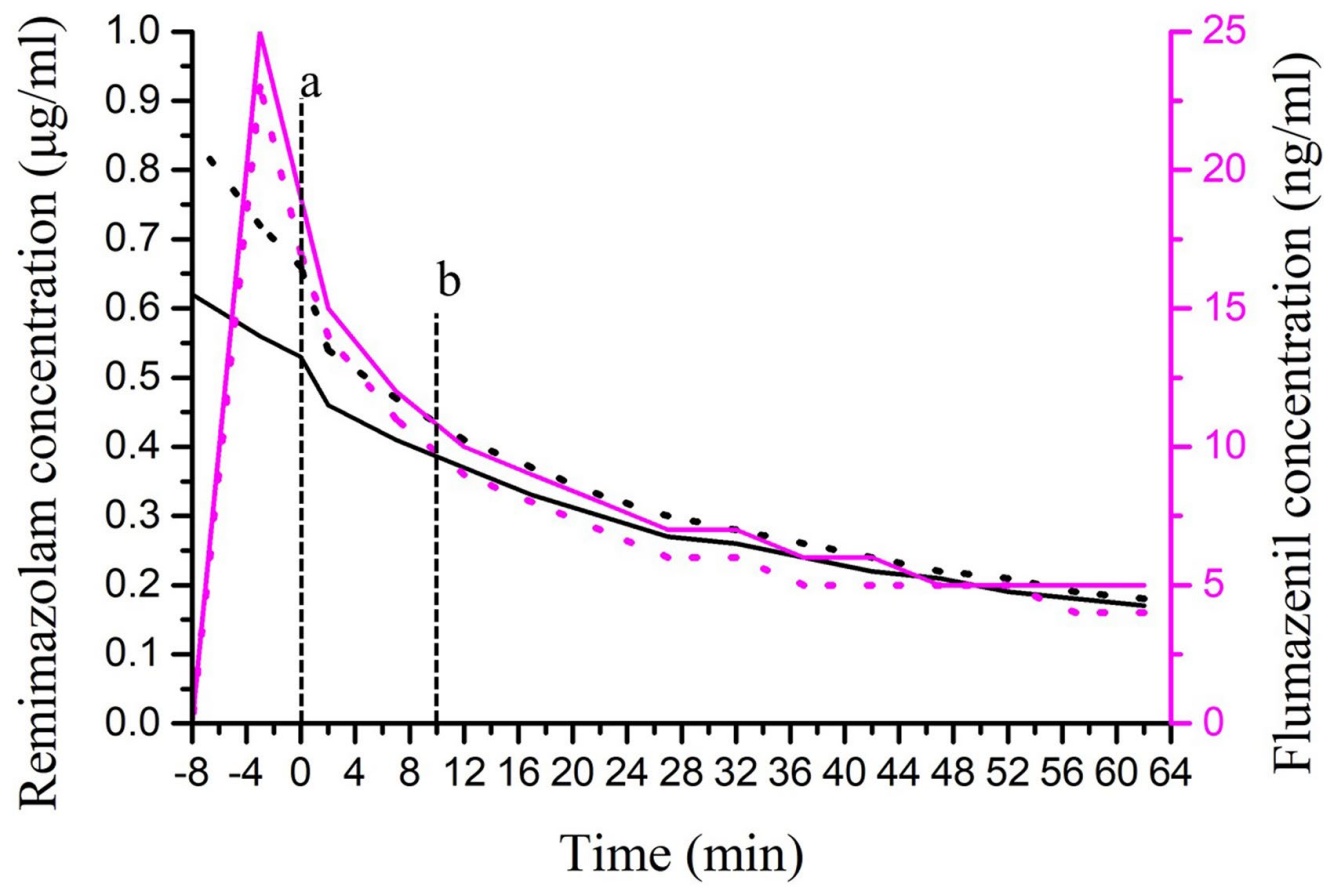
Trends in drug concentrations of remimazolam and flumazenil. The black curve represents the concentration of remimazolam (the black solid line represents the plasma concentration, and the black dotted line represents the effect-site concentration). The purple solid line represents the concentration of flumazenil (the purple solid line represents the plasma concentration, and the purple dotted line represents the brain plasma concentration). Flumazenil (purple curve) is referenced from the right *Y*-axis Vertical dashed lines correspond to case events as follows: (a) The time of extubation. (b) Decline in consciousness noted.

Cases of re-sedation after flumazenil antagonism of remimazolam have been reported (Table 1). In two clinical studies, after a single administration of 0.5 mg of flumazenil for antagonism, 7.1 and 10.8% of the patients experienced re-sedation, respectively (10, 11). In our case, the maintenance dose of remimazolam was 1 mg/kg/h, which strictly adhered to the prescribed protocols. After antagonism with flumazenil, the patient experienced brief awakening and then lost consciousness. No obvious abnormalities were found in imaging examinations or arterial blood gas-analysis. We simulated changes in the concentration of remimazolam using TIVA trainer software. The results indicated that the effect-site concentrations of remimazolam at the end of surgery were 1.11 μg/mL. At the time of extubation, the concentration of remimazolam was 0.72 μg/mL, the brain plasma concentration of flumazenil was 44 ng/mL. At the patient’s level of consciousness declined, the concentration of remimazolam dropped to 0.49 μg/mL (Figure 2), and the brain concentration of flumazenil decreased to approximately 10 ng/mL (Figure 3). As the effect-site concentration of remimazolam approached 0.18 μg/mL, the patient gradually recovered.

**Table 1:** Characteristics of remimazolam re-sedation reported in the literature.

Reference	Surgical procedure	Patients with continuous infusion (n)	Time of re-sedation	Total dose of remimazolam (mg, IQR)	Dose of flumazenil (mg, IQR)	Administration mode of flumazenil	Patients with re-sedation	Time to extubation (mean or median, IQR)
Oh EJ (6)	Catheter ablation for atrial arrhythmia	50	30 min post-procedure	199 (170, 249)	0.4 (0.4, 0.5)	With the return of self-respiration and TOF count of 4, 0.2 mg flumazenil was administered every 2 min; the total dose was ≤ 1 mg	22% (11/50)	6 (4, 8) min
Qiu Y (10)	Endoscopic submucosal dissection	28	NR	N/A	0.5	Immediately after discontinuation of remimazolam, flumazenil 0.5 mg was administered	7.1% (2/28)	5.0 (3.0, 13.8) min
Zhang L (11)	Day surgery	65	30–45 min after remimazolam cessation	82 (55.9, 94.6)	0.5	Five minutes after the end of surgery, flumazenil 0.5 mg was administered	10.8% (7/65)	7.0 (7.0, 8.0) min
Lee B (30)	Robotic gastrectomy	53	NR	322 (244, 391)	0.3 (0.3, 0.3)	When remimazolam was discontinued, 0.2–0.3 mg flumazenil was administered, additional doses were 0.1 mg and the total was ≤ 0.5 mg	2% (1/52)	115 (78, 180) s
Yamamoto T (36)	NR	1	45 min after antagonist	NR	0.5	After remimazolam cessation, 0.5 mg flumazenil was administered	1	NR
Lee SJ (37)	Robotic single-port hysterectomy	1	6 h 35 min after antagonist	160	0.2	Fifteen minutes post-remimazolam discontinuation, 0.2 mg flumazenil was administered	1	NR
Takemori T (38)	Robotic-assisted laparoscopic radical prostatectomy	1	13 min after antagonist	346	0.5	Twenty-eight minutes after remimazolam cessation, 0.5 mg flumazenil was administered	1	23 min

Data are presented as the mean ± SD, number (percentage), or median (interquartile range).

SD, standard deviation; IQR, interquartile range; NR, not reported.

The patient’s physical condition may also have affected their recovery (12). During the second operation, the patient had mild anemia and hypoalbuminemia (hemoglobin 97 g/L, total protein 58.4 g/L, albumin 29.5 g/L). The plasma protein binding of remimazolam is approximately 92%, predominantly to serum albumin (13). The reduction in plasma-bound albumin may increase patient sensitivity (14). Although there is no difference in metabolism among frail patients or those with American Society of Anesthesiologists classification 3+, the steady-state infusion rate should be reduced by 28% (15). Hypoproteinemia may increase the risk of re-sedation.

Remimazolam combined with DEX may prolong a patients’ recovery time (16), only when DEX plasma concentration ≥1.9 ng/mL results in non-wake-up sedation (17, 18). At the end of surgery the plasma concentration of DEX was 0.16 ng/mL (Figure 3). Although hypoalbuminemia can affect the pharmacokinetics of DEX (19), its clearance rate primarily depends on liver blood flow (18, 20). A high hepatic extraction rate may mitigate the effect of altered protein binding on DEX clearance (21). Sedation is not significantly different between patients with mild hypoalbuminemia and normoalbuminemia (20).

### 4.1 Pharmacokinetics

The effect of flumazenil disappeared within 44 min (22, 23). The residual sedative effects of remimazolam indicate that, after 180 min of continuous infusion, the context-sensitive decrement time for an 80% reduction in the effect-site concentration is 44 min (23). Only when the effect-site concentration of remimazolam was less than 0.2 μg/mL, administration of flumazenil could avoid the occurrence of re-sedation (7). Competitive antagonism does not alter the concentration-time curve of remimazolam (24). After a single dose of flumazenil, the receptor occupancy of remimazolam decreases and the patient emerges from anesthesia. As flumazenil is metabolized, residual remimazolam may rebind to GABAA receptors, causing re-sedation (7).

The magnitude of the synergistic effects on sedation outcomes between opioids and remimazolam is dependent on the opioid dose (25). The context-sensitive half-time of remifentanil was approximately 3 min and was independent of the duration of infusion (26). The time from discontinuation of remifentanil to the occurrence of re-sedation in the patient was as long as 18 min. Therefore, we do not consider that re-sedation is related to remifentanil. But coadministration of remifentanil inhibited the apparent elimination clearance of the remimazolam metabolite, CNS7054 (27). CNS7054 may be related to the tolerance to remimazolam, and the occurrence of re-sedation requires further investigation.

### 4.2 Flumazenil timing

The dose of flumazenil used to reverse remimazolam depends on gender, height, weight, and body mass index (28). The administration of flumazenil should be adjusted according to the patient’s response. It is difficult to ascertain whether re-sedation occurs in any particular patient. The recommended initial dose of flumazenil to counteract benzodiazepine-induced sedation is 0.2 mg, followed by an additional 0.2 mg every minute for a total dose of no more than 1 mg (29). However, even with a split-dose regimen, re-sedation may occur because of differences in the timing of flumazenil administration. In two clinical studies, flumazenil administered in split doses immediately after discontinuation of remimazolam, re-sedation occurred in 2 and 22% of patients, respectively (6, 30). In another study, flumazenil was administrated in split doses 10 min after drug discontinuation, and no re-sedation occurred within 60 min (31). The plasma half-life of remimazolam is approximately 4.9 min (23). Delaying the administration of flumazenil may reduce the occurrence of re-sedation. It should be noted that this result may also be owing to the difference in pharmacokinetics between remimazolam tosylate and remimazolam besylate.

### 4.3 Specific risk factors

Remimazolam is metabolized by the non-specific esterase carboxylesterase (CES1), and the genetic polymorphism of CES1 can lead to individual differences in pharmacokinetics (32, 33). The half-maximum effect site concentration of remimazolam ranges from 456 to 934 ng/mL (34). Moreover, the efficiency of carboxylesterase hydrolysis reactions in women is higher than that in men (35). Re-sedation following flumazenil administration has been reported with varying onset and duration. For instance, one case occurred as early as 45 min after a 0.5 mg dose (36), while another emerged 6 h after a 0.2 mg dose (37). In a more prolonged case, re-sedation lasted for 12 h after 0.5 mg of flumazenil was administered 28 min post remimazolam withdrawal (38). Variations in drug metabolism levels may lead to delayed and prolonged re-sedation.

Moreover, the allosteric coupling of the GABAA receptor may lead to the development of rapid tolerance to remimazolam. This tolerance may lead to an increase in the required dosage of remimazolam, as allosteric coupling reduces the ability of benzodiazepines to produce inhibitory post-spike potentials by 50% in the absence of significant changes in the density or affinity of benzodiazepine drug binding sites (39). However, the allosteric coupling could be reversed by flumazenil (40). Therefore, we hypothesized that after flumazenil restored the sensitivity of the GABAA receptor, even a low concentration of remimazolam could lead to re-sedation. In the delayed and prolonged re-sedation in the above cases, this receptor re-sensitization mechanism may have played a significant role.

### 4.4 Clinical recommendations

After antagonism, patients should be monitored promptly to detect the occurrence of re-sedation. To reduce the occurrence of re-sedation, a single high dose of flumazenil should be avoided, delayed administration should be considered, and hypoalbuminemia patients should be closely monitored. Adjusting the dosage of remimazolam depending on the BIS value may lead to overdose and increase the risk of re-sedation (41, 42). Real-time pharmacokinetic simulation can help to aid in adjusting the anesthesia administration time (43).

In the article, some limitations should also be highlighted. First, detailed demographic and clinical characteristics of affected patients were not consistently collected, making it impossible to summarize the characteristics of individuals who are prone to re-sedation. Second, the drug concentrations reported were obtained via software simulation, which may deviate from the actual concentrations. Nevertheless, these simulations align with previously proposed pharmacokinetic characteristics of re-sedation to some extent.

## 5 Conclusion

Re-sedation is an important complication after administering remimazolam and flumazenil in combination. Patients with hypoproteinemia or weakness require extra vigilance. In clinical practice, flumazenil should be used with caution, and administration of a single large dose should be avoided. Determining the gene type of CSE1 may help further clarify the mechanism of re-sedation. Further research is needed to determine the optimal time to administer flumazenil and to identify the demographic and clinical characteristics of patients who experience re-sedation, thereby guiding patient safety.

## Data Availability

The raw data supporting the conclusions of this article will be made available by the authors, without undue reservation.
