# Supplementary material for: Re-sedation using remimazolam anesthesia in patients with multiple injuries during recovery: a case report and literature review
Source: Front Med (Lausanne). 2026 Jan 7;12:1702891. doi: 10.3389/fmed.2025.1702891 (PMC12819805; doi:10.3389/fmed.2025.1702891)

Supplementary Material

# Supplementary Tables

**Supplementary Table 1** The laboratory test results of the patient.

| **Item** | **Result** | **Unit** | **Reference value** |
| --- | --- | --- | --- |
| White blood cell | 5.73 | 10^9^/L | 3.8–10 |
| Red blood cell | 3.33 | 10^12^/L | 3.5–5.5 |
| Hemoglobin | 97 | g/L | 110–165 |
| Blood platelet | 260 | 10^9^/L | 80–300 |
| Total bilirubin | 9.9 | μmol/L | 4.20–22.00 |
| Total protein | 58.4 | g/L | 60.00–85.00 |
| Albumin | 29.5 | g/L | 35.00–55.00 |
| Globulin | 28.9 | g/L | 20.00–35.00 |
| Plasma proteins A/G | 1.02 |  | 1.50–2.50 |
| Alanine transaminase | 44 | U/L | 0–40 |
| Aspartate transaminase | 29 | U/L | 0–40 |
| Ureophil | 5.5 | mmol/L | 2.8–8.2 |
| Creatinine | 36 | μmol/L | 44.0–115.0 |
| Uric Acid | 138 | μmol/L | 90–360 |
| K^+^ | 3.88 | mmol/L | 3.50–5.30 |
| Na^+^ | 142.3 | mmol/L | 135.0–145.0 |
| Cl^+^ | 106.2 | mmol/L | 96.0–11.0 |
| Total serum calcium | 2.08 | mmol/L | 2.10–2.90 |
| Total cholesterol | 3.21 | mmol/L | 2.50–5.20 |
| Creatine kinase | 203 | U/L | 26–140 |
| Creatine kinase MB isoenzyme | 8 | U/L | 0–24 |
| Blood glucose | 5.39 | mmol/L | 3.90–6.10 |
| High-sensitivity C-reactive protein | 73.23 | mg/L | 0.00–6.00 |

# Supplementary Figures


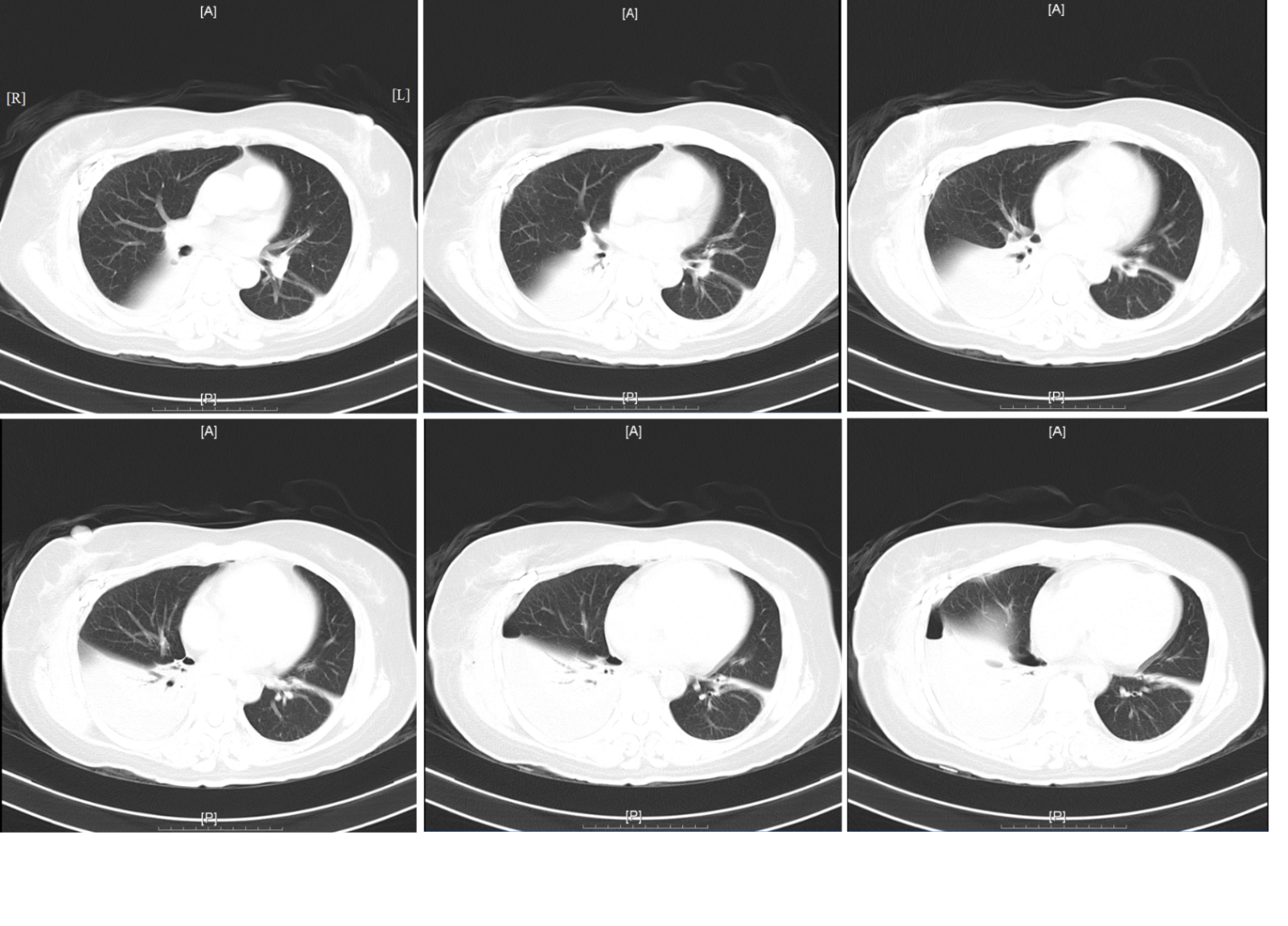


**Supplementary Figure 1.** Chest CT of the patient with atelectasis.


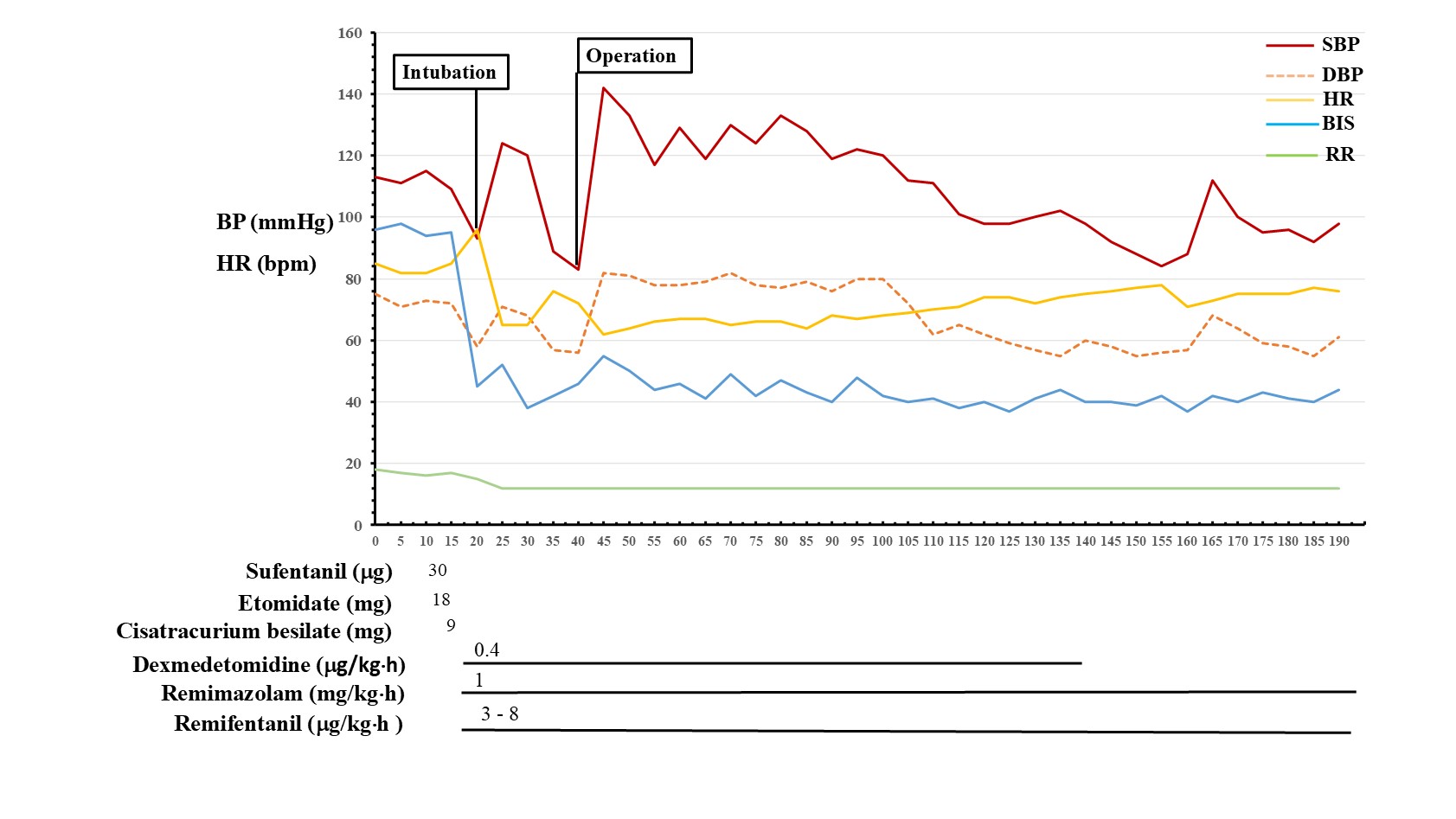


**Supplementary Figure 2.** Anesthesia note.


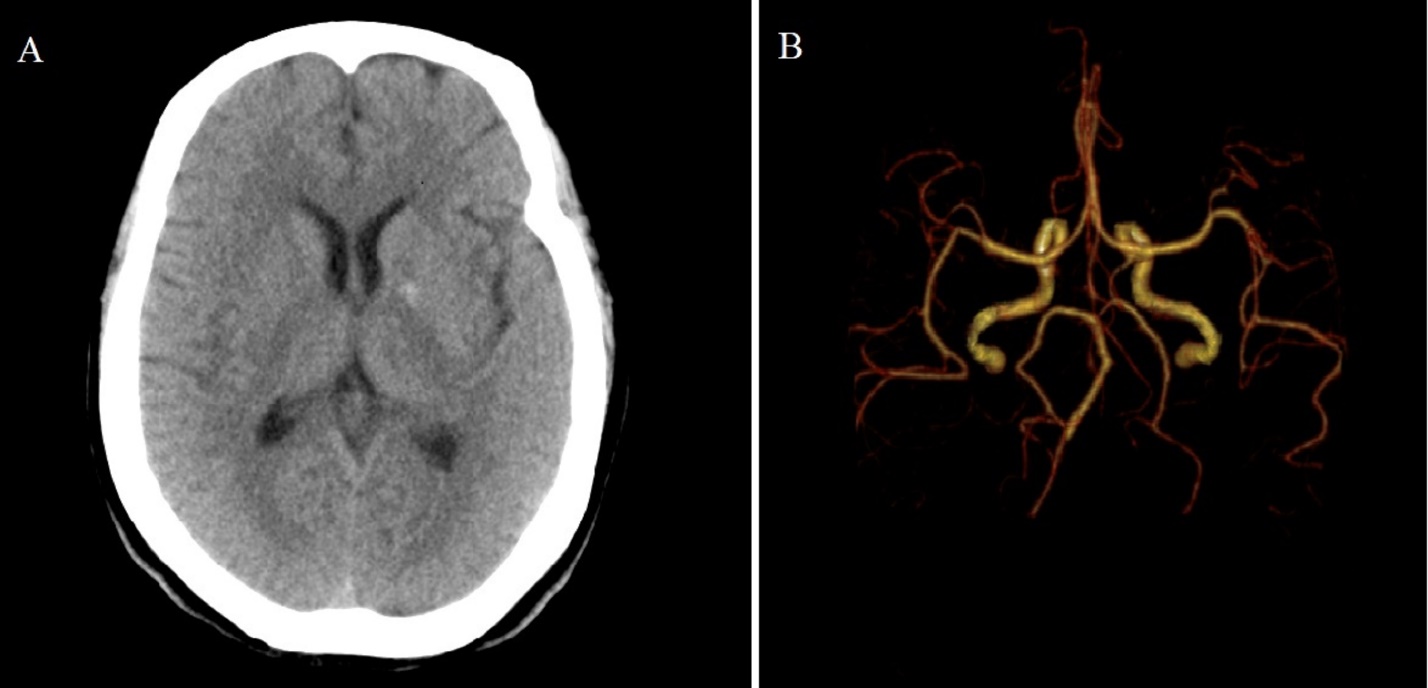


**Supplementary** **Figure 3.** Brain computed tomography and computed tomography angiography.

# Patient informed consent


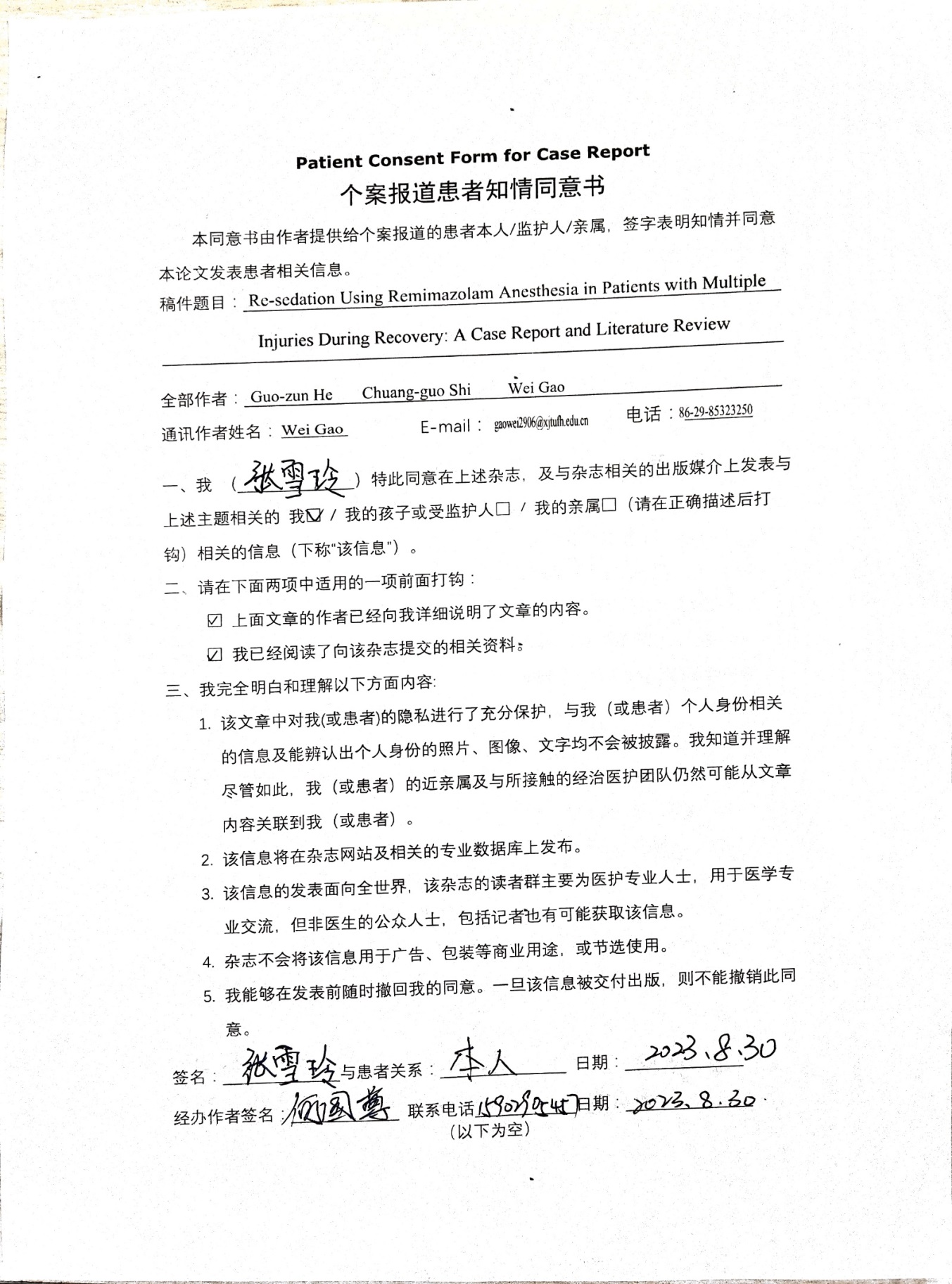

Supplement: Supplementary file 1 [file Supplementary_file_1.docx]
