# Supplementary material for: Re-sedation using remimazolam anesthesia in patients with multiple injuries during recovery: a case report and literature review
Source: Front Med (Lausanne). 2026 Jan 7;12:1702891. doi: 10.3389/fmed.2025.1702891 (PMC12819805; doi:10.3389/fmed.2025.1702891)
Supplement: Supplementary file 2 [file Supplementary_file_2.docx]

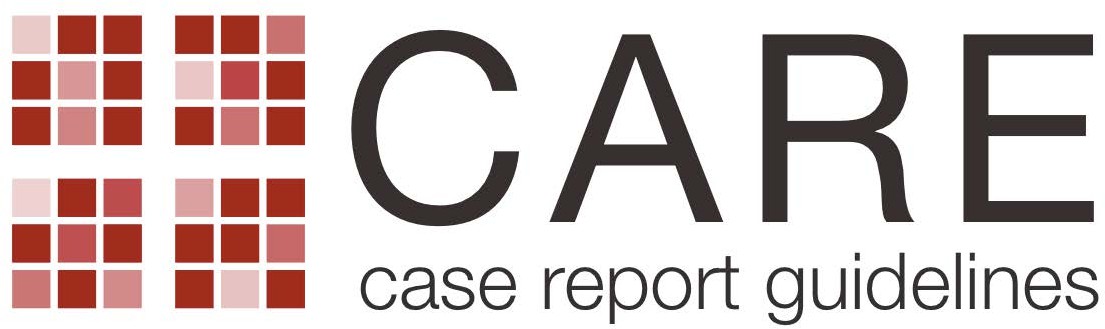
CARE Checklist of information to include when writing a case report
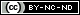


**Title 1** The diagnosis or intervention of primary focus followed by the words “case report” . . . . . . . . . . . . . . . . . . . . . . . . . . 1

**Topic Item Checklist item description Reported on Line**

**Key Words 2** 2 to 5 key words that identify diagnoses or interventions in this case report, including "case report" . . . 1

**Abstract**

**(no references)**

**3a** Introduction: What is unique about this case and what does it add to the scientific literature? . . . . . . . . . . . . . . . . 2

**3b** Main symptoms and/or important clinical findings . . . . . . . . . . . . . . . . . . . . . . . . . . . . . . . . . . . . . . . . . . . . . . . . . . . . . . . 2

**3c** The main diagnoses, therapeutic interventions, and outcomes . . . . . . . . . . . . . . . . . . . . . . . . . . . . . . . . . . . . . . . . . . . 2

**3d** Conclusion—What is the main “take-away” lesson(s) from this case? . . . . . . . . . . . . . . . . . . . . . . . . . . . . . . . . . . . . . 2

**Introduction 4** One or two paragraphs summarizing why this case is unique (**may include references**) . . . . . . . . . . . .

**Patient Information 5a** De-identified patient specific information. . . . . . . . . . . . . . . . . . . . . . . . . . . . . . . . . . . . . . . . . . . . . . . . . . . .

**5b** Primary concerns and symptoms of the patient . . . . . . . . . . . . . . . . . . . . . . . . . . . . . . . . . . . . . . . . . . . . . . . . . . **5c** Medical, family, and psycho-social history including relevant genetic information . . . . . . . . . . . . . . . . . **5d** Relevant past interventions with outcomes . . . . . . . . . . . . . . . . . . . . . . . . . . . . . . . . . . . . . . . . . . . . . . . . . . . . . . . .

4

5

5

6

N/A

**Clinical Findings**

**Timeline**

**Diagnostic Assessment**

**Therapeutic Intervention**

**Follow-up and Outcomes**

1. Describe significant physical examination (PE) and important clinical findings. . . . . . . . . . . . . . . . . . . . . . .
2. Historical and current information from this episode of care organized as a timeline . . . . . . . . . . . . . . .

**8a** Diagnostic testing (such as PE, laboratory testing, imaging, surveys). . . . . . . . . . . . . . . . . . . . . . . . . . . . . . .

**8b** Diagnostic challenges (such as access to testing, financial, or cultural) . . . . . . . . . . . . . . . . . . . . . . . . . . . . .

**8c** Diagnosis (including other diagnoses considered) . . . . . . . . . . . . . . . . . . . . . . . . . . . . . . . . . . . . . . . . . . . . . . . . .

**8d** Prognosis (such as staging in oncology) where applicable . . . . . . . . . . . . . . . . . . . . . . . . . . . . . . . . . . . . . . . **9a** Types of therapeutic intervention (such as pharmacologic, surgical, preventive, self-care) . . . . . . . . . . . . . . . . . . **9b** Administration of therapeutic intervention (such as dosage, strength, duration). . . . . . . . . . . . . . . . . . . . . . . . . . . . **9c** Changes in therapeutic intervention (with rationale) . . . . . . . . . . . . . . . . . . . . . . . . . . . . . . . . . . . . . . . . . . . . . . . . . . . .

**10a** Clinician and patient-assessed outcomes (if available) . . . . . . . . . . . . . . . . . . . . . . .. . . . . . . . . . . . . . . . . . . . . . . . . . . .

**10b** Important follow-up diagnostic and other test results . . . . . . . . . . . . . . . . . . . . . . . . . . . . . . . . . . . . . . . . . . . . . . . . . . . .

**10c** Intervention adherence and tolerability (How was this assessed?) . . . . . . . . . . . . . . . . . . . . . . . . . . . . . . . . . . . . . . . . .

**10d** Adverse and unanticipated events . . . . . . . . . . . . . . . . . . . . . . . . . . . . . . . . . . . . . . . . . . . . . . . . . . . . . . . . . . . . . . . . . . .

6

6

7

6、7

7

7

7

7

7

7

N/A

N/A N/A

**Discussion 11a** A scientific discussion of the strengths AND limitations associated with this case rep . . . . . . . . . . . . . . . . . . . . . . . **11b** Discussion of the relevant medical literature **with references**.. . . . . . . . . . . . . . . . . . . . . . . . . . . . . . . . . . . . . . . . **11c** The scientific rationale for any conclusions (including assessment of possible causes). . . . . . . . . . . . . . . . . . . . . . . **11d** The primary “take-away” lessons of this case report (without references) in a one paragraph conclusion . . . . . . .

**Patient Perspective 12** The patient should share their perspective in one to two paragraphs on the treatment(s) they received. . . . . . .

8

13−17

8−11

12

N/A

**Informed Consent 13** Did the patient give informed consent? Please provide if requested . . . . . . . . . . . . . . . . . . . . . . . . . . . . . . . . . . . . . . **Yes No**

√
